# Pets, protected animals and farm animals: three perceptual spaces of animal abuse

**DOI:** 10.3389/fpsyg.2025.1571336

**Published:** 2025-06-03

**Authors:** Andrea Vera, Ana M. Martín, Stephany Hess-Medler, Bernardo Hernández

**Affiliations:** ^1^Departamento de Psicología Cognitiva, Social y Organizacional, Universidad de La Laguna, La Laguna, Spain; ^2^Departamento de Psicología Clínica, Psicobiología y Metodología, Universidad de La Laguna, La Laguna, Spain

**Keywords:** animal abuse, environmental crime, human-animal studies, green criminology, wild fauna, pets, farm animals, multidimensional scaling

## Abstract

Increased public and political attention to animal abuse has led to changes in legislation that recognize animals as sentient beings. Abusing animals is legally classified in Spain as an environmental crime against flora, fauna and protected areas. Consequently, research into human-animal relationships and animal abuse has also increased. Stereotypes about animals influence how humans treat them. The aim of this study is to analyze the similarities and differences of the perceptual spaces that people spontaneously construct when considering the abuse of protected animals, pets and farm animals, and then to compare them with the space of a prototypical environmental crime such as illegal dumping. Participants were 528 men and women aged between 18 and 88 years old, mostly resident in a highly environmentally protected territory. They completed an online questionnaire containing scenarios, based on press releases, of the four categories of environmental crime. Each participant was randomly asked to rate the scenarios from one of these categories in terms of severity, justification, indignation, intentionality, punishment, likelihood of personal intervention and calling the police. The questionnaire also included questions on socio-demographic data and a social desirability scale. Data were analyzed using multidimensional scaling and the results showed that a three-dimension solution was the best for the four perceptual spaces. However, the content, label and order in which each dimension emerged in the shaping of each space varied. Most pet abuse scenarios were perceived as highly reprehensible and deliberate, with the abuse of dogs and cats being more unjustified and deserving personal intervention than of other companion animals. Scenarios involving the abuse of protected and farm animals elicited less consistent reactions, influenced by the perception of their instrumentality for humans, such as for food or profit. The comparison with illegal dumping suggests that animal abuse is an environmental crime, but with specific characteristics. In contrast with other environmental crimes, its victims are sentient beings and the harm they suffer is both observable and immediate. Future research should explore, in diverse samples and territories, the key variables for effective interventions to prevent and control the social problem of animal abuse.

## 1 Introduction

The debate on animal abuse is having an increasing social and political impact that is reflected in the visibility of the animal rights movement in the media worldwide. The European Parliament for example has revised the legal status of animals to consider them as sentient beings instead of objects or properties^1^, leading some EU countries to modify their laws that protect animals^2^. In Spain in particular, criminal law now protects not only wild fauna but also other categories of animals. Spanish legislative changes began in 2021 with the approval of the *Ley Orgánica 17/2021*, which amended the Civil Code, the Mortgage Act and the Civil Procedure Act to change the legal status of animals by establishing that they are no longer goods but sentient beings whose rights must be protected. Before this change, the Spanish Criminal Code had categorized animal abuse in Title XVI together with other offenses against the environment, including in Chapter III offenses against natural resources and in Chapter IV offences against flora and fauna and the natural environment (arts. 332–336; 338–340). Subsequently, in 2023*, Ley Orgánica 3/2023* amended the Criminal Code adding Title XVIbis offenses against animals and aimed at animals that are domestic, domesticated or living temporarily or permanently under human control. The same year, *Ley 7/2023* was approved to protect and guarantee the rights and welfare of companion and wild animals in captivity.

Despite these legal advances, the Spanish Criminal Code does not give the same legal status to all animals, since animal abuse is only punishable if it is committed “outside of legally regulated activities” (art. 340bis). Indeed, the *Ley Orgánica 7/2023* does not apply to animals used in bullfighting, farm animals, animals used for experimental and other scientific purposes, including teaching, animals used in veterinary clinical research, wild animals not kept in captivity, animals used for sporting or professional activities, as well as hunting dogs, hunting packs and hunting aids animals (art.1). The Spanish public also seems to differentiate between categories of animals when asked about animal’s capacity to suffer and feel pain, their status within the family and the punishment people convicted of animal abuse deserve. In this case, the distinction is made based on how emotionally close and attached people feel to the animal (Bernuz and María, 2022).

The visibility of the animal rights movement in the media has also contributed to research questioning the human-animal relationships and focusing on human behaviors that harm animal welfare (Bègue and Vezirian, 2024; Hodson and Dhont, 2023). In the scientific literature, animal abuse is defined as “nonaccidental, socially unacceptable behavior that causes pain, suffering or distress to and/or the death of an animal” (Ascione and Shapiro, 2009, p. 570). Human emotions and behaviors toward animals depend on stereotypes, defined as socially shared beliefs about different categories of animals (Sevillano and Fiske, 2020). From an intergroup perspective approach, Sevillano and Fiske (2016) applied the Stereotype Content Model (Fiske et al., 2002) and the Behaviors from Intergroup Affect and Stereotypes Map (Cuddy et al., 2007) to analyze how people categorize animals. Sevillano and Fiske (2016) consider that this categorization relies on attributing similarities to animals on dimensions such as the perception of competence, which is viewed as intelligence, and the perception of warmth, understood as friendliness toward humans. In relation to the perception of competence, humans tend to preserve animals they consider skillful whereas they ignore those they feel are unskillful. When it comes to the warmth dimension, animals perceived as friendly will elicit protective care, while those perceived as unfriendly may lead to violent responses of self-defense. Based on these attributions, the authors label animals perceived high in competence and low in warmth as “predators” (e.g., lions, tigers), those perceived high in competence and warmth as “companions” (e.g., dogs, cats), those perceived low in competence and high in warmth as “prey” (e.g., pigs, cows), and those perceived low in competence and warmth as “pests” (e.g., rats, snakes). Predators would elicit awe, active harm, and passive help; companions, fondness and active and passive help; prey, indifference, passive harm, and active help; and pests, contempt and passive and active harm (Sevillano and Fiske, 2016, 2019).

Other authors have classified animals according to several criteria, such as the attribution of mind (Leach et al., 2021, 2023), attractiveness (Collado et al., 2022), wildness (Dhont and Hodson, 2020), instrumentality as food (McGuire et al., 2023) or as experimental subjects (Bègue and Vezirian, 2024), as well as their legal considerations (Martín et al., 2023). It is worth noting that the attribution to animals of characteristics such as having minds has been used to justify the quality and legitimacy of human relationships with them (Leach et al., 2021, 2023). Whereas, the perception of animals’ lack of mental capacities has been used to legitimize their instrumental use by humans (Bastian and Loughnan, 2017; Loughnan and Davies, 2020; Leach et al., 2021, 2023; Potocka and Bielecki, 2023). The traditional status of animals in social sciences has been that of human property, and until recently even green criminology has considered animal abuse a minor offence, always in term of harm to the owner and not to the animal as a sentient being (Beirne, 2011).

The legal considerations of animals as sentient beings, the legal status of different categories of animals, and the legal classification of animal abuse as an environmental offense, make the Spanish legal context very suitable for studying public perception of animal abuse. Taking advantage of this research setting, Vera et al. (2023) focused on farm animals to analyze the relationship between public reaction against their abuse and attitudes toward animals in general. Likewise, Martín et al. (2023) compared pets and protected animals in terms of bystanders’ reactions to their abuse and its relation to personality traits. However, there is no research that simultaneously assesses how the general public spontaneously perceives the three legal categories of animals: wild fauna (herein protected animals), pets and farm animals.

Previous research on environmental offenses studied scenarios of animal abuse as offenses against the environment, including offenses against wild flora and fauna, as well as offences against natural resources (e.g., Martín et al., 2011; Martín et al., 2013a,b; Martín et al., 2014). Martín et al. (2011), for example, showed that people spontaneously classify scenarios of environmental offenses into three types: offenses against the natural environment (protected flora, fauna and natural areas), illegal construction activities and illegal dumping (natural resources). They found that people perceived scenarios of offences against natural environment and of illegal dumping as more serious, deserving harsher punishment, generating more indignation and more unjustified than illegal construction activities. The differences between scenarios of offences of illegal dumping and against the natural environment (protected flora, fauna and natural areas) is that illegal dumping is perceived as generating more profits, being more frequent, and depending on public authorities for their control.

Martín et al. (2011) also provided information on the perceptions of the specific scenarios of environmental offences, regardless of the categorization made by the authors or the participants. Their results showed that the participants rated most negatively the scenario of the local council allowing sewage from a housing estate to be discharged into the ocean, as well as the scenario of the hunter who shot and killed a kestrel, a protected species. These results are in line with other studies carried out at different times and with different samples (Hernández et al., 2005; Martín et al., 2008, 2013a,b). When discussing their results, Martín et al. (2013a) pointed out that future research should address the question of whether the more negative rating of these specific scenarios could be due to the kestrel and the ocean being associated with participants’ social identity, or that the two scenarios were equally related to biodiversity. In Martín et al. (2011), the authors analyzed scenarios involving animals other than the kestrel, which was always explicitly identified as wild fauna such as dolphins being chased by a tourist boat or hunting dogs abandoned by their owner at the end of the hunting season. The scenarios involving animals were included in the category of offenses against the natural environment, along with scenarios of offenses against wild flora, such us cutting down a protected dragon tree or uprooting a population of endangered endemic orchids. Other offenses included those against protected natural areas such as driving across a nature reserve, camping illegally on a beach or burning stubble during the peak fire warning season. The study design allowed for comparisons between specific scenarios of animal abuse (kestrel, dolphins and hunting dogs). However, it did not make comparisons between abuse of different categories of animals, such as protected animals, pets and farm animals.

To fill these gaps, this study aims to explore how people perceive animal abuse depending on the Spanish legal category to which the victim belongs. The perceptual spaces spontaneously constructed when representing the abuse of protected animals, pets and farm animals are analyzed and compared using multidimensional scaling. In addition, given that animal abuse is an environmental offense in legal terms, the similarities and differences between these perceptual spaces and those constructed by people in relation to illegal dumping are examined. Illegal dumping has been selected for comparison because it is an environmental offense included in the Title XVI of offenses against the environment, as Chapter III on offenses against the natural resources. Also, previous research has shown that this type of offense is perceived as negatively and as generating same levels of rejection as animal abuse (Hernández et al., 2005; Martín et al., 2008, 2011, 2013a,b). The damage caused by illegal dumping, such as the contamination of water and marine life or the contamination of natural spaces, often cause irreparable damage. Thus, it is well known and generates great concern among the people in the study setting (Martín et al., 2013a,b).

Considering animal abuse as an environmental offense, from both a legal and psychosocial perspective, has the advantage of allowing research to focus on offenses rather than offenders, using variables that have already been studied in relation to compliance with environmental laws within a broader psychosocial paradigm (Martín et al., 2014). This starting point is consistent with an approach to human-animal conflict that takes into account the values associated with nature, in terms of utilitarianism versus conservation, domination versus subjugation, rural versus urban lifestyles, rather than competition for space, resources or life (Sevillano and Fiske, 2020; Treves, 2008). From this perspective, what people use to differentiate categories of animals may not be the magnitude of the damage they may cause or their conservation status, but their power to elicit strong mixed opinions from different sectors of society, often leading to confrontations between groups of people who hold different values toward these animals and their management (Marchini, 2014). Although this is the first study to simultaneously compare independent perceptual spaces of abuse of different categories of animals, it is expected from previous research on environmental offenses and human-animal relationships that there will be similarities between the perceptual spaces of animal abuse and of illegal dumping. However, it is also anticipated that there will be more similarities among the perceptual spaces of the abuse of the three categories of animal than between them and the space of illegal dumpling.

## 2 Materials and methods

### 2.1 Participants

The study originally enrolled 763 Spanish-speaking participants. Those who did not complete the entire questionnaire and/or had a high social desirability score (>14; Gutiérrez et al., 2016) were excluded in order to clean the data matrix. The social desirability score was used to control that participants’ responses reflected their authentic opinions and not a desire to conform to social conventions, as animal abuse can be considered a controversial topic. The final sample was 528 participants, half men and half women, ranging in age between 18 and 88 years old (*M* = 30.17; *SD* = 13.56), mostly residents in the Canary Islands (95.6%). There were 59.1% living in an urban area, 27.3% in a rural area, and 13.6% in a coastal area of the same territory, which is highly protected by environmental law. The level of education of participants was 41.7% university studies, 29.9% high school, vocational training 20.2%, secondary school 5.5 and 3.1% had not completed primary school. Regarding employment status, 52.1% were students, 40.9% were employed, 4.9% were unemployed and 2.1% were pensioners.

### 2.2 Instruments

A questionnaire was prepared to include three sections: Sociodemographic data, scenarios related to environmental crimes and the Marlowe-Crowne Social Desirability Scale.

#### 2.2.1 Sociodemographic data

Participants were asked about sociodemographic data such as gender, age, area of residence (urban, rural, or coastal), educational level, and employment status.

#### 2.2.2 Scenarios related to environmental offenses

Forty scenarios, based on press releases, were selected to describe situations of four types of environmental offences. Ten scenarios referred to illegal dumping (e.g., “A company discharges pollutants down a drain into a ravine”), and the other thirty referred to animal abuse, distributed equally among protected animals (e.g., “A fishing boat catches a loggerhead turtle that is close to shore.”), pets (e.g., “A group of young people left a hamster they had at home on the street”), and farm animals (e.g., “A couple keep pigs crammed into crates in unhygienic conditions”).

Press reports were used to look for scenarios of animal abuse and illegal dumping, as they would be more familiar to study participants than those described in court records. Twenty press reports for each type of offense, covering a variety of situations and involving different actors, were selected from several local newspapers available online. Subsequently, a group of experts in Environmental and Legal Psychology discussed the situations in the press reports in terms of their relevance for the study, having occurred in the study setting and whether it was likely that most people would be familiar with them. As a result of experts’ comments, six situations were excluded from each type of offenses. The remaining 14 press reports of each type were used to write scenarios describing the situations concisely, eliminating unpleasant and unnecessary details, but making them credible so they could be imagined by the reader. During the writing process, special attention was paid to who the perpetrator was, which animal was abused, and the consequences of the offence. Afterwards, a court judge verified the illegality of all the situations described in the scenarios, both of animal abuse and illegal dumping.

To select the final scenarios, a pilot study was carried out with 25 experts in Environmental Psychology. The suitability of the scenarios was checked by asking these experts to rate each scenario on an 11-point Likert-type scale (0–10) as to whether they could imagine the situation, whether they thought the situation was representative of anti-environmental behavior, whether the situation was realistic enough to have occurred in their immediate environment, and whether it was written in inclusive language. Based on an analysis of the data from this pilot study, four scenarios from each category were rejected and the remaining 40 were retained for the study (see Supplementary Appendix).

Each participant was randomly asked to rate 10 scenarios of one of the four types of offenses on an 11-point Likert-type scale (from 0 to 10), based on the severity, justification (unjustified), indignation, intentionality, the punishment they would assign it, and the probability of personal intervention and calling the police.

#### 2.2.3 Marlowe-Crowne social desirability scale

Marlowe-Crowne social desirability scale (Crowne and Marlowe, 1960) was applied in its reduced version from the Spanish adaptation of Gutiérrez et al. (2016). This scale is used to control biased responses by participants as it measures the tendency of participants to respond in a socially appropriate way through 18 items (e.g., “I always try to practice what I preach”) with dichotomous responses (0 = “False” and 1 = “True”), which are summed to obtain a final score. Gutiérrez et al. (2016) provide evidence of validity and reliability, with an internal consistency measured by Cronbach’s alpha of 0.78. In this study, McDonald’s omega was 0.64.

### 2.3 Procedure

The questionnaire was administered online using the Qualtrics^XM^ platform, with the collaboration of students from the Psychology, Social Work and Law degrees at the Universidad de La Laguna, who received extra points in one subject as compensation. Following the snowball technique, students distributed the link to acquaintances, reaching people of different genders, ages and areas of residence. In the questionnaire instructions, participants were informed that the information collected in the questionnaire would be used exclusively for research and scientific publication purposes. In addition, the anonymous and confidential nature of their responses was guaranteed and express consent to participate was requested before they could begin answering the questionnaire. The presentation of the scenarios and response scales was randomized to avoid carry-over effects. The procedure respected the ethical principles of the Declaration of Helsinki and was approved by the Comité de Ética de la Investigación y Bienestar Animal de la Universidad de La Laguna (CEIBA2022-3220).

### 2.4 Data analysis

Multidimensional scaling with individual differences was carried out for each type of scenario, using the Proxcal v.10 of the SPSS v.26 statistical package. The input matrixes were computed by averaging the squared differences of the scores of all participants in each pair of transgressions on each scale for each type of scenario [see Forgas et al. (1980) for a more detailed description of the procedure]. Each input matrix corresponded to each scale and reflected the differences between each pair of transgressions for each type of scenario. This procedure generates outputs which include the coordinates of each transgression, as well as the individual weights of each scale, for each dimension of the scaling and for each type of scenario. These scale weights represent very useful quantitative information for interpreting these dimensions. Means of each scenario in each scale were also used to support the interpretation of dimensions.

## 3 Results

The results of the four multidimensional scaling carried out for the 10 scenarios of each of the three types of animal abuse and illegal dumping from the seven rating scales are described below, following the Forgas procedure (Forgas et al., 1980; Forgas, 1982). The first space is the one for protected animals, the second for pets, the third for farm animals and the last for illegal dumping.

### 3.1 Protected animals

**Figure 1:**
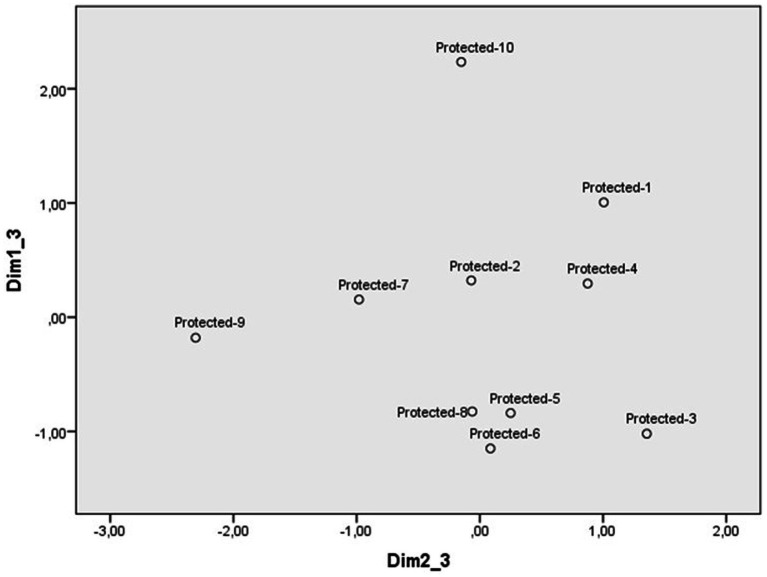
Dimensions 1 (Reprobation) and 2 (Intentionality) of the perceptual space of protected animal abuse scenarios.

**Figure 2:**
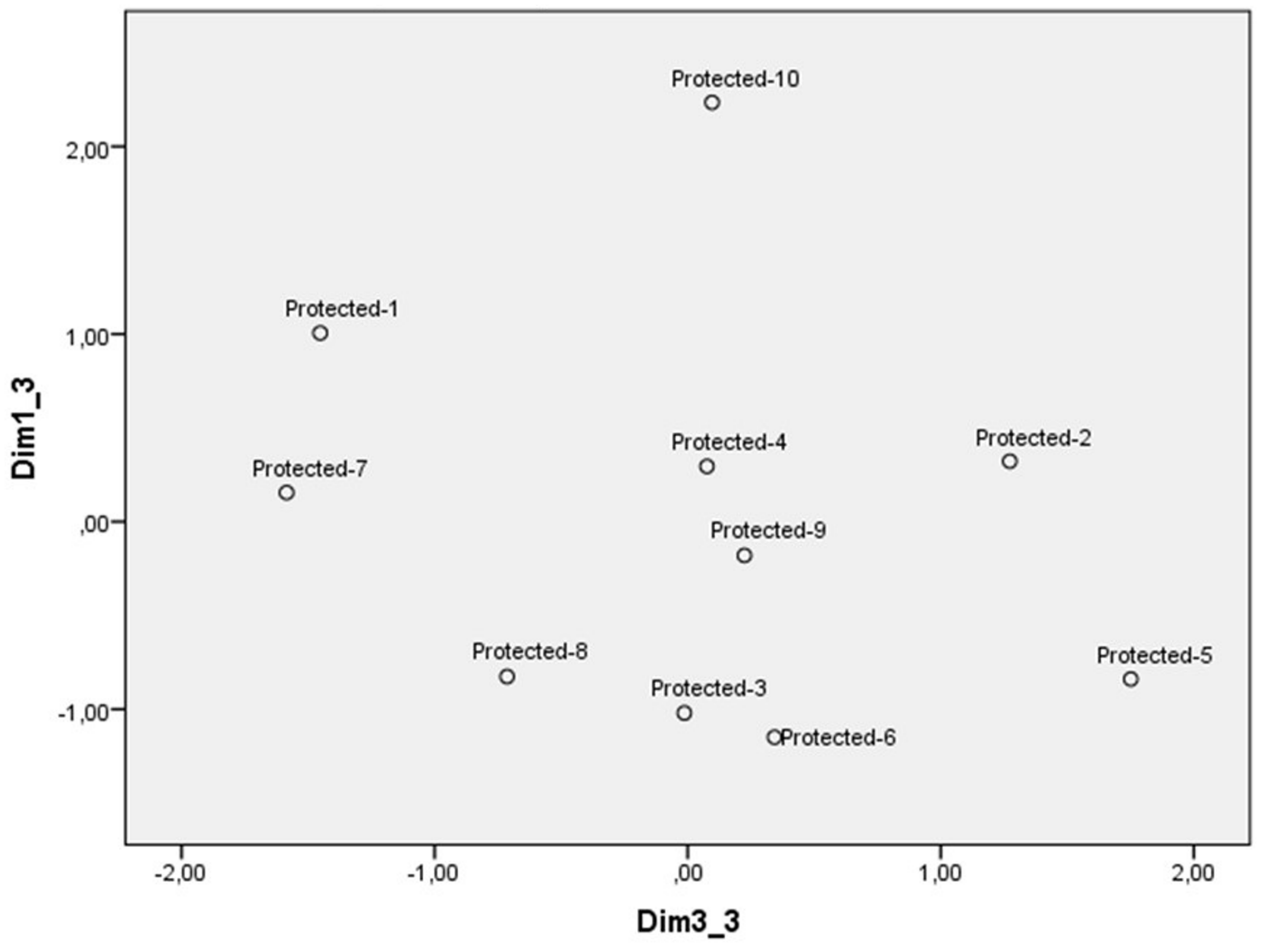
Dimensions 1 (Reprobation) and 3 (Reaction) of the perceptual space of protected animal abuse scenarios.

Scaling of protected animal abuse scenarios from the evaluation scales identified a three-dimensional perceptual space. Kruskal’s standardized raw stress was 0.05. The weights of each scenario in dimensions 1 and 2 are reflected in Figure 1, those of dimensions 1 and 3 in Figure 2, and those of the scales in Table 1. The first dimension contrasts the scenario of a couple throwing stones at a long-eared owl to make noise, injuring one of its wings with the scenario in which a group of people hunt several cory’s shearwaters to make a casserole. As the scales of greatest weight in this dimension are (non)justification, punishment, indignation and severity, this dimension can be labeled reprobation. Participants perceive as more reprehensible the scenarios at the bottom of Figure 1, which they value as the most unjustified, indignant, serious, and deserving of punishment, compared to those at the top, considered as the least reprehensible.

**Table 1:** Weighting of the seven scales according to the perceptual dimensions of abuse scenarios of protected animals.

Scales	Dimension		
	1	2	3
Severity	0.446	0.359	0.333
(Non)justification	0.492	0.358	0.201
Indignation	0.456	0.387	0.276
Intentionality	0.260	0.500	0.314
Personal intervention	0.424	0.292	0.387
Punishment	0.480	0.319	0.322
Call the police	0.343	0.261	0.494

The second dimension (Figure 1) places, at one extreme, the scenario of a fishing boat that catches a loggerhead turtle that is close to shore and, at the other, the scenario of a couple who feed poisoned food to a blue chaffinch nesting in their window. The scale with the greatest weight is that of intentionality, so this dimension has been labeled intentionality. Although participants consider all the behaviors described in the scenarios intentional (see Table 2), they locate on the right side of Figure 1 those scenarios considered the most intentional. Only the scenario related to the loggerhead turtle is rated as less intentional and located at the opposite extreme.

**Table 2:** Means (standard deviation) of each scale for each scenario of protected animal abuse.

	Scales						
Scenarios	Severity	(Non)Justification	Indignation	Intentionality	Personal Intervention	Punishment	Call the police
Protected-1	8.14 (2.53)	8.44 (2.57)	8.30 (2.50)	8.35 (2.51)	5.12 (3.51)	7.41 (2.91)	5.35 (3.79)
Protected-2	8.83 (1.71)	9.09 (1.78)	8.71 (1.91)	9.16 (1.79)	5.69 (3.20)	8.24 (2.30)	6.27 (3.48)
Protected-3	9.02 (1.88)	9.16 (2.01)	9.30 (1.66)	8.85 (2.56)	7.11 (3.15)	8.55 (2.28)	6.69 (3.29)
Protected-4	8.55 (2.25)	8.93 (2.18)	8.67 (2.13)	8.94 (1.98)	5.91 (3.23)	8.00 (2.73)	6.27 (3.44)
Protected-5	9.49 (1.48)	9.58 (1.15)	9.27 (1.74)	9.30 (1.92)	6.62 (3.30)	9.27 (1.70)	8.20 (2.38)
Protected-6	9.03 (1.71)	9.42 (1.67)	9.23 (1.67)	9.08 (2.02)	7.64 (2.71)	8.64 (2.12)	6.55 (3.24)
Protected-7	8.12 (2.44)	8.94 (2.01)	8.19 (2.48)	8.77 (2.36)	5.13 (3.48)	7.88 (2.65)	5.88 (3.62)
Protected-8	8.71 (1.89)	9.46 (1.27)	8.91 (1.81)	8.57 (2.43)	6.96 (3.00)	8.16 (2.49)	5.72 (3.67)
Protected-9	8.44 (2.48)	8.86 (2.19)	8.17 (2.76)	7.74 (3.08)	6.22 (3.36)	7.73 (2.91)	6.70 (3.44)
Protected-10	7.83 (2.82)	8.09 (2.69)	7.80 (2.89)	8.91 (2.16)	5.45 (3.60)	7.06 (3.40)	5.75 (3.78)

The third dimension (Figure 2) contrasts the scenarios of a gang selling a Canary eagle by posting an ad on social networks and a hunter killing a kestrel by shooting it with his shotgun during a hunt, with the scenarios of a pleasure boat aggressively chasing a pod of sperm whales during an excursion and a fishing boat capturing a monk seal to keep its fins as a trophy. The scales that carry the most weight is personal intervention and calling the police, so this dimension has been labeled as a reaction to transgression. It is necessary to point out that the scale of personal intervention defines this dimension, but it also carries weight in the dimension of reprobation. On the extreme right are the scenarios in which the participants are more willing to intervene in some way, until they reach the extreme left, where their willingness to act is lower, although they continue to show interest in intervening (see Table 2).

### 3.2 Pets

**Figure 3:**
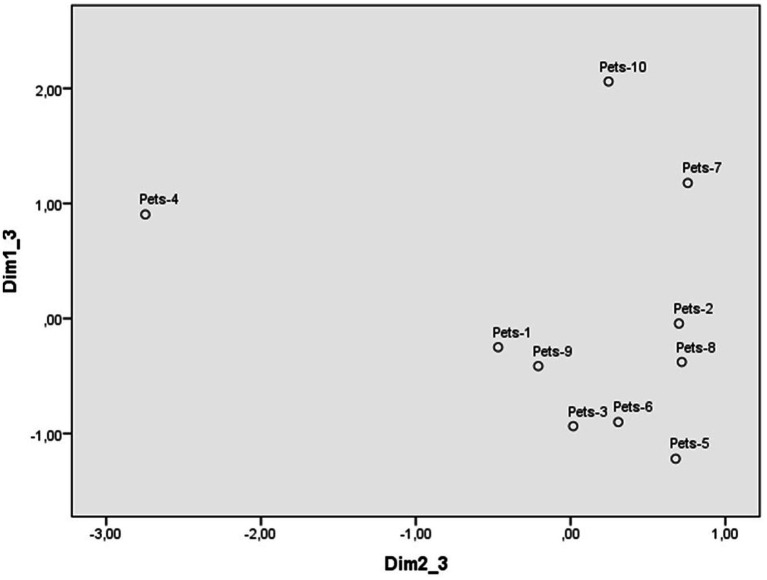
Dimensions 1 (Reprobation) and 2 (Intentionality) of the perceptual space of pet abuse scenarios.

**Figure 4:**
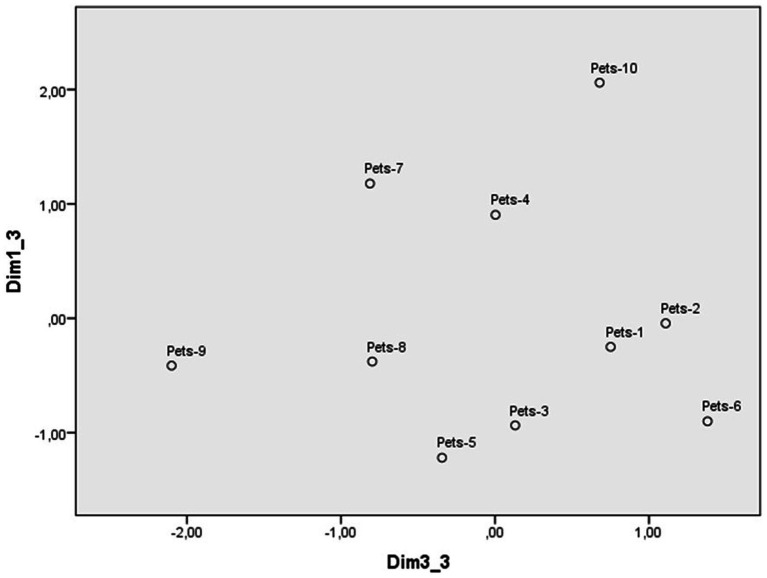
Dimensions 1 (Reprobation) and 3 (Justification) of the perceptual space of pet abuse scenarios.

Scaling of the pet abuse scenarios from the evaluation scales also allowed the identification of a three-dimensional perceptual space, with a Kruskal standardized raw stress of 0.04. The weights for each scenario in dimensions 1 and 2 are reflected in Figure 3, those for dimensions 1 and 3 in Figure 4, and those for the scales in Table 3.

**Table 3:** Weighting of the seven scales according to the perceptual dimensions of abuse scenarios of pets.

Scales	Dimension		
	1	2	3
Severity	0.566	0.300	0.160
(Non)justification	0.381	0.347	0.400
Indignation	0.562	0.252	0.232
Intentionality	0.242	0.571	0.223
Personal intervention	0.369	0.379	0.370
Punishment	0.480	0.409	0.209
Call the police	0.514	0.256	0.310

The first dimension (Figure 3) contrasts the scenario of a group of young people who left a hamster they had at home on the street with the scenario of neighbors who suffocated a stray dog that was found near their house. The scales with the strongest weight in this dimension were severity, indignation, calling the police, and punishment, so this dimension has been labeled as reprobation. Participants perceive the scenarios at the bottom of Figure 3 as more reprehensible, which are rated as more serious, indignant, deserving of punishment and being reported to the police. In comparison, those higher up are rated less negatively, even though those at the upper end are still reprehensible.

The second dimension (Figure 3) places at one extreme the scenario in which a person lets his/her cat die because he/she has not taken it to the vet to have an injury treated, compared to those of several friends who film themselves plucking a bird and upload it to social media and of a person who kicks and throws a rabbit several meters that has approached them in the bush. The scale with the greatest weight is intentionality, so this dimension was labeled intentionality. As for protected animals, participants considered all the behaviors intentional that were described in the scenarios on pet abuse (see Table 4). They placed all the scenarios on the extreme right of Figure 3, as the most intentional, and only the scenario in which a person lets his/her cat die on the extreme left, as the least intentional.

**Table 4:** Mean (standard deviation) of each scale for each scenario of pet abuse.

	Scales						
Scenarios	Severity	(Non)Justification	Indignation	Intentionality	Personal Intervention	Punishment	Call the police
Pet-1	9.45 (1.39)	9.55 (1.42)	9.37 (1.49)	9.08 (2.44)	7.16 (3.28)	9.06 (1.85)	8.11 (2.86)
Pet-2	9.39 (1.41)	9.48 (1.73)	9.42 (1.29)	9.33 (1.89)	7.71 (2.98)	8.83 (2.10)	7.57 (3.33)
Pet-3	9.70 (0.85)	9.69 (1.24)	9.71 (1.03)	8.90 (2.43)	8.58 (2.43)	9.33 (1.69)	8.91 (1.96)
Pet-4	8.80 (1.75)	8.83 (2.11)	8.90 (1.90)	7.36 (3.01)	6.83 (3.40)	7.68 (3.03)	5.00 (4.00)
Pet-5	9.78 (0.76)	9.51 (1.18)	9.65 (1.30)	9.36 (2.03)	8.33 (2.65)	9.50 (1.57)	8.81 (2.40)
Pet-6	9.73 (0.73)	9.48 (1.85)	9.68 (1.26)	9.25 (2.20)	7.43 (3.16)	9.51 (1.26)	8.32 (3.17)
Pet-7	9.04 (1.66)	9.51 (1.40)	9.03 (2.13)	9.09 (1.99)	7.42 (2.98)	8.27 (2.44)	5.44 (3.71)
Pet-8	9.57 (1.01)	9.70 (1.09)	9.53 (1.36)	9.36 (2.04)	7.67 (3.14)	9.20 (1.63)	7.17 (3.43)
Pet-9	9.46 (1.25)	9.39 (1.92)	9.50 (1.51)	9.11 (2.37)	7.14 (3.23)	8.77 (2.28)	6.27 (3.86)
Pet-10	8.71 (2.03)	9.27 (1.94)	8.81 (2.29)	8.95 (2.47)	6.88 (3.42)	7.89 (2.90)	4.98 (3.78)

The third dimension (Figure 4) contrasts the scenario of a dog breeder who cuts the vocal cords of his dogs to prevent them from making any noise, with the scenario of a person who cuts off a bird’s beak so it cannot sing because the noise disturbs them. The scales with the most weight were (non)justification and personal intervention, so this dimension was labeled justification. The majority of the abuse scenarios are located in the right half of Figure 4, as the participants consider them to be unjustified and deserving of personal intervention. The four scenarios that are placed on the left side of the figure are also considered unjustified but slightly less so. The victim of abuse in two cases is a bird and in the third a rabbit, while those on the other end are always cats and dogs, including hunting dogs (see Table 4).

### 3.3 Farm animals

**Figure 5:**
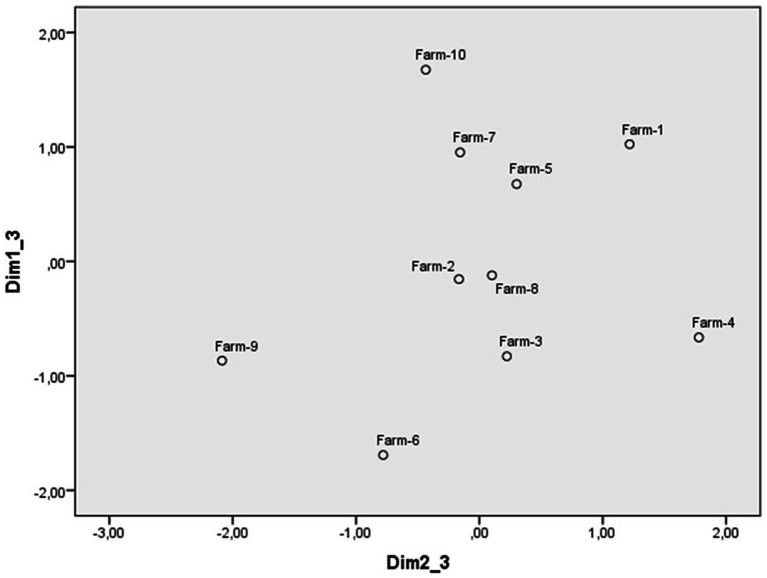
Dimensions 1 (Reaction) and 2 (Reprobation) of the perceptual space of scenarios of farm animal abuse.

**Figure 6:**
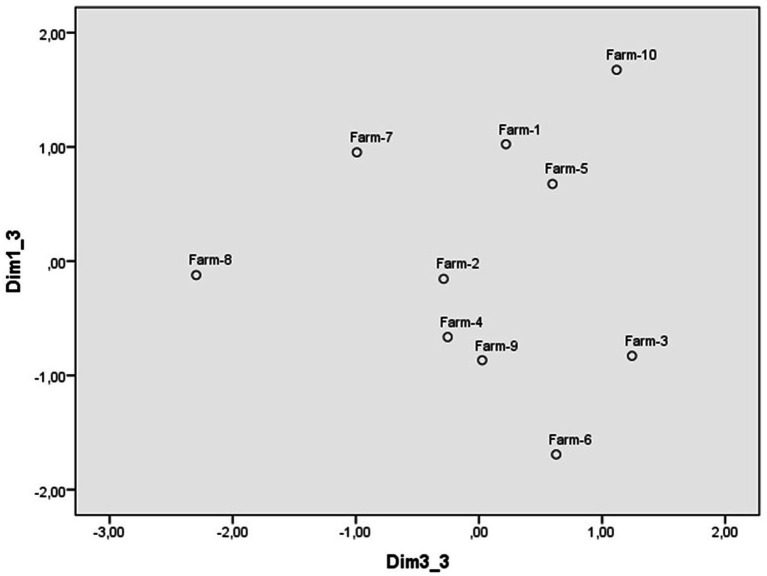
Dimensions 1 (Reaction) and 3 (Intentionality) of the perceptual space of scenarios of farm animal abuse.

The scaling of the scenarios of abuse of animals raised for human consumption from the evaluation scales also led to the identification of a three-dimensional perceptual space with a Kruskal standardized raw stress of 0.05. The weights of each scenario in dimensions 1 and 2 are reflected in Figure 5, those of dimensions 1 and 3 in Figure 6, and those of the scales in Table 5. The first dimension (Figure 5) contrasts the scenario in which a person uses fast-fattening methods on chickens, with that of a person who ties his sheep to a tree and leaves them in the sun without food or water. The scales with the highest weights in this dimension are personal intervention, calling the police and (non)justification, so this dimension was named reaction to transgressions. At the upper extreme are the scenarios to which people are more willing to intervene in some way, considering them unjustified, to those at the lower extreme that elicit less reaction. It is interesting to note that the animals at this extreme are birds and fish that are usually consumed directly as food, while those at the other extreme are used for various purposes (see Table 6).

**Table 5:** Weighting of the seven scales according to the perceptual dimensions of abuse scenarios of farm animals.

Scales	Dimension		
	1	2	3
Severity	0.431	0.443	0.221
(Non)justification	0.429	0.377	0.266
Indignation	0.361	0.484	0.248
Intentionality	0.233	0.255	0.558
Personal intervention	0.560	0.191	0.270
Punishment	0.403	0.369	0.366
Call the police	0.517	0.331	0.254

**Table 6:** Mean (standard deviation) of each scale for each scenario of farm animal abuse.

	Scales						
Scenarios	Severity	(Non)Justification	Indignation	Intentionality	Personal intervention	Punishment	Call the police
Farm-1	9.02 (2.11)	8.90 (2.40)	9.20 (1.76)	7.91 (3.00)	6.49 (3.53)	8.64 (2.46)	8.20 (2.57)
Farm-2	8.76 (2.15)	8.80 (2.13)	8.68 (2.29)	7.55 (3.07)	5.53 (3.61)	8.17 (2.47)	7.34 (3.17)
Farm-3	8.63 (2.14)	8.63 (2.22)	8.48 (2.53)	7.88 (2.94)	5.01 (3.53)	7.92 (2.70)	6.62 (3.56)
Farm-4	8.73 (2.27)	8.81 (2.18)	8.73 (2.46)	7.98 (2.97)	5.61 (3,71)	7.78 (2.89)	6.88 (3.47)
Farm-5	9.21 (1.77)	8.95 (2.19)	9.05 (2.12)	7.92 (2.92)	6.44 (3.41)	8.72 (2.16)	7.80 (3.86)
Farm-6	8.47 (2.44)	8.39 (2.46)	8.45 (2.43)	8.75 (2.45)	4.95 (3.41)	7.79 (2.82)	6.02 (3.57)
Farm-7	9.16 (1.78)	9.09 (1.89)	9.02 (1.94)	7.13 (2.96)	6.79 (3.25)	8.57 (2.22)	7.67 (2.92)
Farm-8	8.78 (2.17)	8.84 (2.02)	8.68 (2.40)	5.86 (3.44)	5.98 (3.45)	7.82 (2.87)	7.16 (3.50)
Farm-9	8.21 (2.53)	8.41 (2.35)	8.14 (2.73)	8.00 (2.78)	4.64 (3.43)	7.61 (2.81)	6.12 (3.76)
Farm-10	9.12 (2.09)	9.13 (1.66)	9.32 (1.66)	8.52 (2.62)	7.47 (3.40)	8.81 (2.31)	8.26 (2.74)

The second dimension (Figure 5) puts at one extreme the scenario in which workers on a fish farm are overcrowding the ponds and impeding the movement of fish and at the other that of a person who lets cows suffer slowly instead of slaughtering them if calving is not going well. The scales that have a stronger weight in this dimension are indignation, severity, and punishment, which is why it has been labeled as reprobation. In this case, on the extreme right are the animal abuse scenarios that are considered the most reprehensible and on the left the least reprehensible, although all scenarios were rated very negatively.

The third dimension (Figure 6) contrasts the scenario of a couple that do not activate the cooling system on their farm and the hens suffocate in the high temperatures, with the scenario of a person who ties his/her sheep to a tree and leaves them in the sun without food or water, and of the farm owners who overcrowd hens in the same cage without allowing them to move around. The scale with the highest weight was intentionality, so this dimension has also been labeled as intentionality. All scenarios of abuse of farm animals are also rated as intentional, but less than those of protected animals and pets (see Table 6). Participants considered the scenario of not activating the cooling system on their farm to be the least intentional, placing it at the left extreme of Figure 6, and the most intentional at the right extreme, passing through those attributed an intermediate intentionality.

### 3.4 Illegal dumping

**Figure 7:**
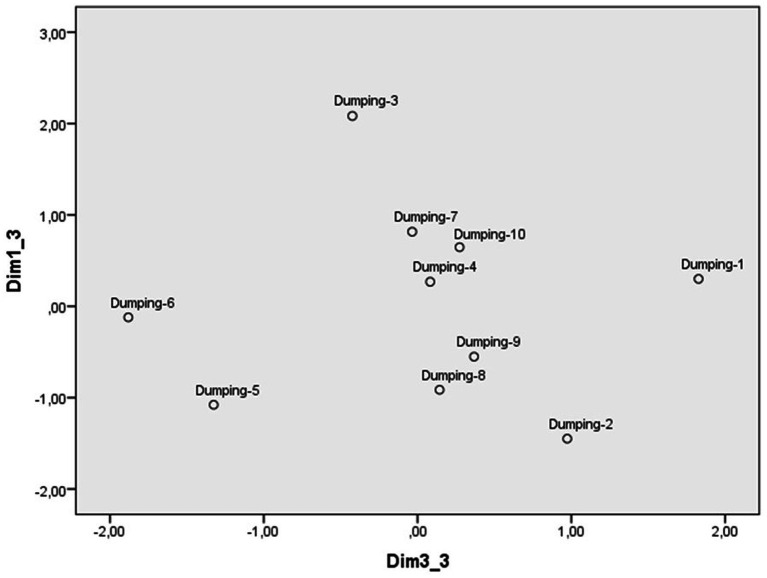
Dimensions 1(Reprobation) and 2(Reaction) of the perceptual space of scenarios of illegal dumping.

**Figure 8:**
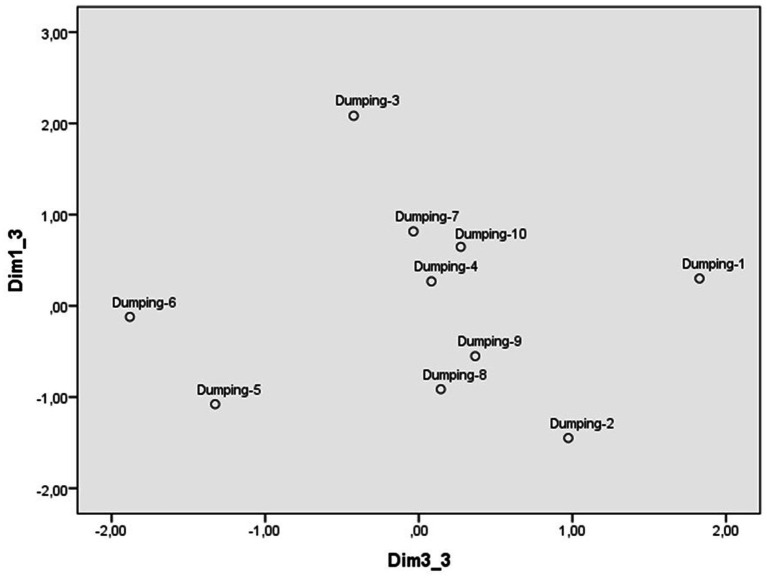
Dimensions 1(Reprobation) and 3(Justification) of the perceptual space of scenarios of illegal dumping.

The scaling of the illegal dumping scenarios from the evaluation scales identified a three-dimensional perceptual space with a Kruskal standardized raw stress of 0.06. The weights of each scenario in dimensions 1 and 2 are reflected in Figure 7, those of dimensions 1 and 3 in Figure 8 and those of the scales in Table 7. The first dimension (Figure 7) contrasts the scenario in which some neighbors dispose of old equipment on some common land with those of a local council that allows poorly treated sewage from a housing estate to be discharged into the sea and a company that discharges pollutants down a drain into a ravine. As Table 4 shows, the scales with the strongest weight in this first dimension were severity, punishment, and indignation, so this dimension was labeled reprobation. Although all the scenarios are valued negatively, those located at the lower extreme are perceived more negatively, compared to those located higher up, until reaching the upper extreme, where those with the least disapproval are located.

**Table 7:** Weighting of the seven scales according to the perceptual dimensions of abuse scenarios of illegal dumping.

Scales	Dimension		
	1	2	3
Severity	0.508	0.279	0.302
(Non)justification	0.344	0.247	0.477
Indignation	0.511	0.262	0.309
Intentionality	0.351	0.386	0.375
Personal intervention	0.360	0.450	0.291
Punishment	0.541	0.295	0.238
Call the police	0.299	0.513	0.265

The second dimension (Figure 7) places at one extreme the scenarios of a person who disposes of used surgical masks by throwing them into a ravine or of a group of young people who leave bags of rubbish on a beach in a nature reserve and, at the other, the scenarios of a person who abandons his/her old car in a ravine after buying a new one or of a company that discharges pollutants down a drain into a ravine. The scales that have the strongest weight in this dimension are personal intervention and calling the police, which is why it has been labeled as a reaction to transgression. The participants place on the extreme left the two scenarios in which they are more inclined to intervene personally, while at the other extreme are those against which they are more inclined to call the police. In the middle are the scenarios in which they could act in one way or the other (see Table 8).

**Table 8:** Mean (standard deviation) of each scale for each scenario of illegal dumping.

Scenarios	Scales						
	Severity	(Non)justification	Indignation	Intentionality	Personal intervention	Punishment	Call the police
Dumping-1	8.79 (1.96)	9.53 (1.35)	8.64 (2.10)	8.67 (2.41)	5.07 (3.41)	8.32 (2.38)	6.99 (3.27)
Dumping-2	9.44 (1.41)	9.51 (1.23)	9.40 (1.35)	8.92 (2.02)	5.37 (3.51)	9.23 (1.56)	6.17 (3.61)
Dumping-3	8.11 (2.37)	9.13 (1.80)	8.34 (2.48)	8.99 (1.99)	5.11 (3.49)	7.48 (2.96)	5.41 (3.62)
Dumping-4	8.79 (1.88)	9.62 (1.08)	9.06 (1.67)	9.04 (2.05)	6.43 (3.25)	8.00 (2.48)	3.61 (3.47)
Dumping-5	9.37 (1.58)	9.25 (1.93)	9.16 (1.68)	8.83 (2.17)	5.09 (3.55)	9.00 (1.82)	6.63 (3.46)
Dumping-6	9.12 (1.74)	9.14 (2.10)	8.95 (2.02)	9.00 (1.83)	6.05 (3.30)	8.63 (2.15)	6.70 (3.67)
Dumping-7	8.66 (2.18)	9.27 (1.67)	8.64 (2.11)	9.06 (1.83)	5.48 (3.28)	8.29 (2.39)	5.46 (3.60)
Dumping-8	9.37 (1.46)	9.43 (1.55)	9.20 (1.68)	9.03 (2.16)	6.08 (3.19)	9.23 (1.47)	7.14 (3.26)
Dumping-9	8.99 (1.70)	9.48 (1.44)	9.30 (1.56)	8.89 (2.10)	7.27 (3.10)	8.38 (2.13)	4.98 (3.51)
Dumping-10	8.76 (1.99)	9.52 (1.04)	8.66 (2.14)	8.89 (2.38)	4.67 (3.38)	8.33 (2.28)	6.74 (3.11)

The third dimension (Figure 8) contrasts the scenario in which some people from the neighborhood are dumping rubbish in a Nature Park with the scenario in which a person abandons his/her old car in a Protected Area. The scales with the strongest weight were justification (unjustified) and intentionality, so this dimension has also been labeled as justification. On the extreme left are the scenarios that the participants consider most unjustified and intentional, and on the right are those perceived as most justifiable and least intentional (Table 8).

## 4 Discussion

The aim of this study is to explore how the general public perceive animal abuse, comparing it to other environmental offences, such as illegal dumping. Also, as emotions and behavior toward animals may vary depending on the type of animal victimized, scenarios of three categories of animals were compared. It was expected that more similarities between the three types of animal abuse than between them and illegal dumping would be found. The results of the multidimensional scaling showed that a three-dimension solution was the best for the four categories of environmental crimes, but that the nature of these dimensions was not the same in all cases.

The procedure followed for the multidimensional scaling on this occasion used as input matrixes the average of the squared differences of the scores assigned to each pair of scenarios by all participants on each scale. The advantage of this procedure over the traditional one (e.g., Cano et al., 2025), in which each input matrix corresponds to one participant, is that in addition to providing the weights of each scenario in the scaling dimensions, it also facilitates the weights of the scales in relation to these dimensions. These weights provide very useful quantitative information for the interpretation of the dimensions that would otherwise have to be based exclusively on the relative proximities of the scenarios.

The four perceptual spaces were defined by three dimensions. However, the content, label and order in which each dimension emerges in the shaping of the perceptual space of each type of transgression deserves some comment. As animal abuse is the focus of this study, the discussion of the similarities and differences between the perceptual spaces of the three categories of animals will be prioritized over comparisons with illegal dumping. The perceptual space that stands out at first glance is the one in which most scenarios of pet abuse are displayed in the lower right side of the figure delimited by the two first dimensions (Figure 1). In contrast, scenarios of protected and farm animals tend to distribute around the central point of the space defined by these dimensions (Figure 3 and Supplementary Figure 1). This distribution suggests that most cases of pet abuse are perceived as highly reprehensible and deliberate, in line with previous research on the close relationship between humans and pets. Indeed, pets have been perceived as high in both competence and warmth, eliciting fondness as well as active and passive help (Sevillano and Fiske, 2016, 2019; Sims et al., 2007). Pets are attributed more intelligence, sentience, capacity to suffer and having minds in general terms, than other types of animals (Leach et al., 2021, 2023). They are seen as similar to humans and are often treated as such (Levitt et al., 2016), with more care and affection than other animals (Caviola et al., 2019), leading some people to find spending time with pets more enjoyable than with other humans (Lades et al., 2020).

The public shows a stronger rejection of harming pets than other animals because they are given a higher status (Caviola et al., 2019). The results of the present study are consistent with previous evidence by showing that there are differences even within the category of pets: the abuse of dogs and cats is seen as more unjustified and deserving personal intervention than that of others animals that people often think of as pets, even though the law does not recognize them as such (e.g., rabbits, birds) (see Figure 4). Whether an animal is a pet or not depends more on social and cultural norms than on its characteristics as a living being (Bailey et al., 2016), even in a legal context. The abuse of protected animals and farm animals is also negatively perceived, but there is more dispersion of scenarios across spaces in terms of people’s reprobation and attributing intentionality to harm. The main difference between these two categories of animals is that the dimension that first emerges in the perception of farm animals is reaction against the transgression, not reprobation. People considered less justified and deserving higher intervention those scenarios of abuse against animals that are not food (McGuire et al., 2023), even when they are used for other human purposes (e.g., horses pulling buggies for tourist rides). This result suggests that the instrumentality of animals for humans plays an important role in the perception of their abuse, not only when comparing protected and farm animals, but also within the category of farm animals. Indeed, the acceptance or rejection of the use of different animals varies according to social and cultural norms and is therefore shaped throughout the lifelong development of individuals (Marchini, 2014; McGuire et al., 2023).

Looking at the perception space of protected animals abuse, it is worth noting that values associated with nature play a role in the definition of human-animal relationships. Different sectors of society may have conflicting values about protected animals and their management, such as conservation versus utilitarianism, subjugation versus domination, rural versus urban lifestyles (Marchini, 2014). From this perspective, what the participants in this study use to differentiate protected animals does not seem to be their conservation status or competition for resources (space, wellbeing, etc.), but whether harming them can become instrumental in generating profits. It is these profits that makes the abuse of protected animals less reprehensible (e.g., “A pleasure boat aggressively chases a pod of sperm whales during an excursion”). In the case of prototypical environmental crimes such as illegal dumping, profits have the opposite effect, making people more likely to disprove of scenarios involving companies or public authorities than those involving individuals. This finding is consistent with research into environmental crimes, which has found that illegal dumping is perceived to generate more profits and to depend more on public authorities for control than transgressions against natural environment, including transgression against flora and fauna (Martín et al., 2011).

Another difference worth noting in the case of the abuse of protected animals is that participants would both call the police and intervene personally, whereas in the case of illegal dumping, the type of intervention depends on the nature of the scenario. In general, people prefer to call the police in cases of major environmental damage, such as the dumping of cars, tires or household appliances, and to intervene personally in less serious cases, such as the dumping of sanitary masks or rubbish on the beach. However, when it comes to reacting to the abuse of protected animals, all possible forms of intervention can be used, maybe because victims are specific living beings rather than a vague and abstract environment. These findings suggest that animal abuse is a specific type of environmental crime and should therefore be studied with its specific characteristics in mind. In general, environmental crimes have consequences that may not be immediate, and their victims are an indeterminate group of people who may be affected in the long term (Martín et al., 2008). In contrast, in the case of animal abuse, the victim is a specific living being and the consequences of the harm it suffers are obvious and immediate. This psychological approach is in line with green criminology’s interest in defining animal abuse and investigating types of animal abuse that are socially acceptable. There is also interest in what categories of animal abuse are reported to authorities, and how speciesism influences human-animal interactions, among other questions (Beirne, 2011).

Summing up, it is possible to conclude that the comparisons made in this study between perceptual spaces can be a first step in understanding people’s perceptions of both the abuse of different categories of animals, in particular, and of environmental crime in general. It is true that this study was carried out in a highly environmentally protected territory, with many endemic species of flora and fauna, where environmental laws are very salient. This can be seen as both a limitation and an advantage, but in any case, this salience may have affected the perception of protected species but not pets and farm animals. A second limitation is that the results are mainly descriptive and causal relationships cannot be established. However, this study contributes knowledge to this field by highlighting the need for future research to carry out intragroup and experimental designs with different samples in different territories, to further investigate the perceptions of the abuse of different animal categories focusing on the behavior rather than the abuser. Clearly, the ultimate aim of this research is to identify the key variables for effective interventions to prevent and control the social problem of animal abuse through human education.

## Data Availability

The raw data supporting the conclusions of this article will be made available by the authors, without undue reservation.

## Footnotes

^1^ https://www.europarl.europa.eu/topics/en/article/20200624STO81911/animal-welfare-and-protection-eu-laws-explained-videos

^2^ https://www.animal-ethics.org/la-situacion-legal-de-los-animales-en-europa/
