# Supplementary material for: Pets, protected animals and farm animals: three perceptual spaces of animal abuse
Source: Front Psychol. 2025 Jun 3;16:1571336. doi: 10.3389/fpsyg.2025.1571336 (PMC12170516; doi:10.3389/fpsyg.2025.1571336)

Supplementary Figure. Dimensions 2 (Reprobation) and 3(Intentionality) of the perceptual space of scenarios of farm animals

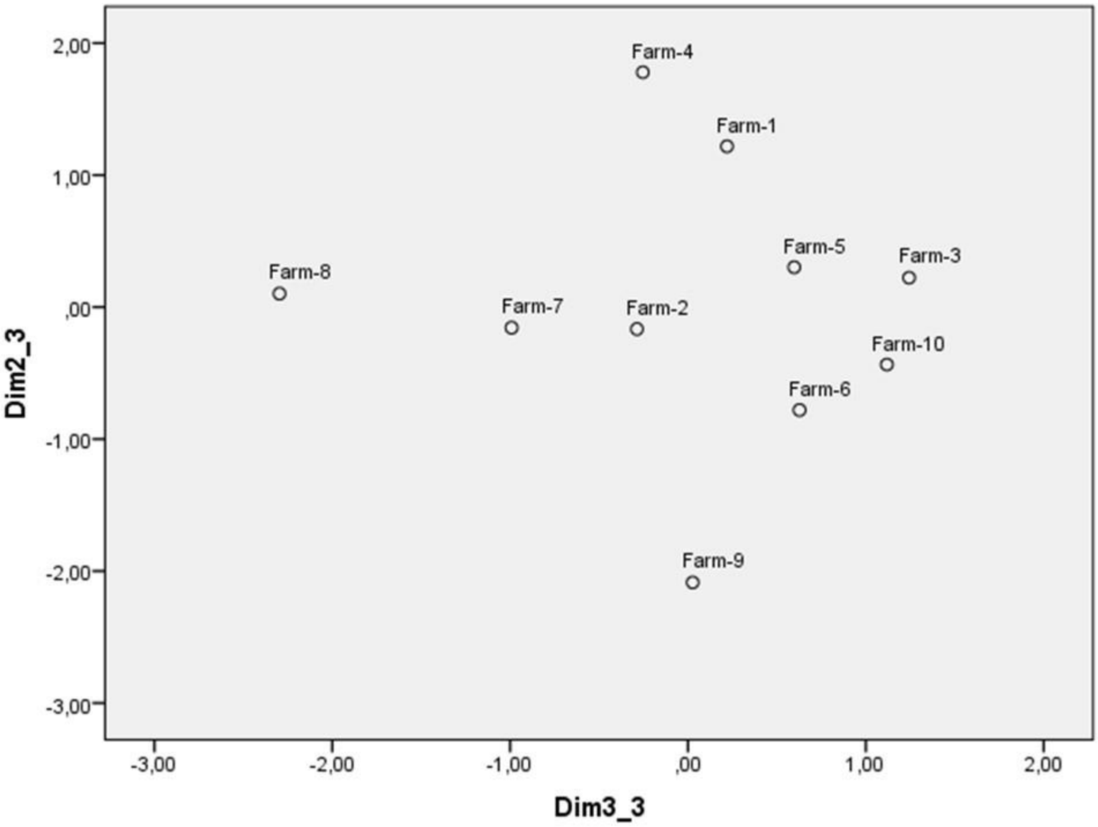

Supplement: Supplementary file 1 [file Image_1.pdf]
