# Supplementary material for: Pets, protected animals and farm animals: three perceptual spaces of animal abuse
Source: Front Psychol. 2025 Jun 3;16:1571336. doi: 10.3389/fpsyg.2025.1571336 (PMC12170516; doi:10.3389/fpsyg.2025.1571336)
Supplement: Supplementary file 2 [file Supplementary_file_1.docx]

Appendix 1. Scenarios of transgression of animal abuse and illegal dumping.

|  | Abuse of Protected Animals | | |
| --- | --- | --- | --- |
|  | Scenarios | Original version | English translation |
|  | Protected-1 | Un cazador mata a un cernícalo disparándole con su escopeta durante una cacería | A hunter kills a kestrel by shooting it with his shotgun during a hunt. |
|  | Protected-2 | Una embarcación recreativa persigue agresivamente a una manada de cachalotes durante una excursión. | A pleasure boat aggressively chases a pod of sperm whales during an excursion. |
|  | Protected-3 | Una pareja da comida envenenada a un pinzón azul que había anidado en su ventana | A couple feeds poisoned food to a blue chaffinch nesting in their window |
|  | Protected-4 | Varios cazadores matan a un cuervo canario que se encontraba posado en un árbol. | Hunters kill a Canary crow perching in a tree |
|  | Protected-5 | Un barco pesquero captura una foca monje para quedarse las aletas como trofeo. | A fishing boat captures a monk seal to keep its fins as a trophy |
|  | Protected-6 | Una pareja tira piedras a un búho chico porque hacía ruido, hiriéndole en un ala. | A couple throws stones at a long-eared owl to make noise, injuring one of its wings. |
|  | Protected-7 | Una banda vende una aguililla canaria poniendo un anuncio a través de las redes sociales. | A gang sells a Canary eagle by posting an ad on social networks |
|  | Protected-8 | Una persona hiere a una pardela tirándole una lata de cerveza mientras planeaba sobre el mar. | A person injures a shearwater bird by throwing a beer can at it as it glides over the sea. |
|  | Protected-9 | Un pesquero captura una tortuga boba que se encontraba cerca de la costa. | A fishing boat catches a loggerhead turtle that is close to shore. |
|  | Protected-10 | Un grupo de personas cazan varias pardelas cenicientas para realizar una cazuela. | A group of people hunt several Cory's shearwaters to make a casserole |
|  | Abuse of Pets | | |
|  | Pet-1 | Varios cazadores abandonan a sus perros en el monte al finalizar la temporada de caza. | Some hunters leave their dogs in the bush at the end of the hunting season |
|  | Pet-2 | Una familia se va de vacaciones y abandona a su gato en una gasolinera de la carretera | A family goes on holiday and leaves their cat at a petrol station on the motorway. |
|  | Pet-3 | Una persona deja sin agua ni comida a sus perros, dejándoles morir en la azotea | A person leaves his dogs without food and water, leaving them to die on the roof |
|  | Pet-4 | Una persona deja morir a su gato por no llevarlo al veterinario para que se le curen una herida | A person lets his cat die because he hasn't taken it to the vet to have an injury treated. |
|  | Pet-5 | Unos vecinos asfixian a un perro callejero que se encontraba cerca de su domicilio | Neighbors suffocated a stray dog that was found near their house. |
|  | Pet-6 | Una persona criadora de perros les corta las cuerdas vocales para que no hagan ruido | A dog breeder cuts the vocal cords of his dogs to prevent them from making any noise. |
|  | Pet-7 | Una persona da una patada y lanza varios metros a un conejo que se le acercó en el monte | A person kicks and throws a rabbit that has approached him in the bush several meters. |
|  | Pet-8 | Varios amigos se graban en vídeo desplumando a un pájaro para subirlo a redes sociales | Several friends film themselves plucking a bird and upload it to social media. |
|  | Pet-9 | Una persona le corta el pico a su pájaro para que no pueda cantar porque le molesta el ruido | A person cuts off a bird's beak so it can't sing because the noise disturbs it. |
|  | Pet-10 | Unos jóvenes abandonan en la carretera a un hámster que tenían en casa. | A group of young people left a hamster that they had at home on the street. |
|  | Abuse of farm animals | | |
|  | Farm-1 | Una persona tiene a sus caballos en una cuadra junto a otros caballos muertos | A person has his horses in a stable with other dead horses |
|  | Farm-2 | Un matrimonio tiene cerdos hacinados en jaulas en condiciones insalubres | A couple keep pigs crammed into crates in unhygienic conditions |
|  | Farm-3 | Los dueños de una granja hacinan un número excesivo de gallinas en una misma jaula sin que pueda moverse | Farm owners overcrowd hens in the same cage without allowing them to move around. |
|  | Farm-4 | Una persona deja agonizar lentamente a sus vacas, en lugar de sacrificarlas, cuando el parto no llega a buen término | A person lets his cows suffer slowly instead of slaughtering them if calving is not going well |
|  | Farm-5 | Una persona deja encerradas a sus cabras en un corral sin agua ni alimentos | A person leaves his goats locked in a pen without food and water |
|  | Farm-6 | Una persona utiliza procedimientos para el engorde rápido de pollos y aumentan tanto de peso que no pueden moverse | A person uses fast-fattening methods on chickens, and they put on so much weight that they can't move |
|  | Farm-7 | Los dueños de una carreta no protegen a su caballo de las altas temperaturas y termina desplomándose en la calle | The owners of a cart do not protect their horse from the high temperatures, and it collapses in the street |
|  | Farm-8 | Una pareja no activa el sistema de refrigeración de su granja y las gallinas se mueren asfixiadas por las altas temperaturas | A couple does not activate the cooling system on his farm and the hens suffocate in the high temperatures |
|  | Farm-9 | Los trabajadores de una piscifactoría hacinan un número excesivo de peces en los estanques impidiendo su movimiento | Workers on a fish farm are overcrowding the ponds and impeding the movement of fish |
|  | Farm-10 | Una persona ata a sus ovejas a un árbol y las abandona al sol sin agua ni comida | A person ties his sheep to a tree and leaves them in the sun without food or water |
|  | Illegal Dumping | | |
|  | Dumping-1 | Una persona abandona su coche viejo en un espacio declarado Paraje Protegido. | A person abandons his old car in a Protected Area. |
|  | Dumping-2 | Un ayuntamiento permite que las aguas fecales, mal depuradas, de una urbanización se viertan al mar | A local council allows poorly treated sewage from a housing estate to be discharged into the sea. |
|  | Dumping-3 | Algunas personas del vecindario se deshacen de electrodomésticos viejos en un solar del barrio | Some neighbors dispose of old equipment in a neighborhood land |
|  | Dumping-4 | Una persona se deshace de sus mascarillas quirúrgicas usadas tirándolas a un barranco. | A person disposes of his used surgical masks by throwing them into a ravine. |
|  | Dumping-5 | Una empresa vierte sustancias contaminantes por un desagüe que da a un barranco | A company discharges pollutants down a drain into a ravine |
|  | Dumping-6 | Algunas personas del vecindario tiran escombros en un lugar declarado Parque Natural | Some people from the neighborhood are dumping rubbish in a declared Nature Park |
|  | Dumping-7 | . La persona encargada de un taller se deshace del aceite de coche usado en un terreno cercano | The manager of a garage disposes of used car oil in a nearby field. |
|  | Dumping-8 | Un taller de repuestos desecha los neumáticos viejos tirándolos en una playa cercana | A tire shop disposes of old tires by dumping them on a nearby beach |
|  | Dumping-9 | Un grupo de jóvenes deja unas bolsas con basura en una playa de una Reserva Natural | A group of young people leave bags of rubbish on a beach in a nature reserve. |
|  | Dumping-10 | Una persona abandona su coche viejo en un barranco tras comprarse uno nuevo | A person abandons his old car in a ravine after buying a new one |
